# View Order With Arbitrary Center Echoes

**DOI:** 10.1002/mrm.70482

**Published:** 2026-06-18

**Authors:** Henric Rydén, Ola Norbeck, Sophie Schauman, Mikael Skorpil, Adam van Niekerk

**Affiliations:** ^1^ Department of Neuroradiology Karolinska University Hospital Stockholm Sweden; ^2^ Department of Clinical Neuroscience Karolinska Institutet Stockholm Sweden; ^3^ Department of Molecular Medicine and Surgery Karolinska Institutet Stockholm Sweden

**Keywords:** FSE, MPRAGE, MRI, RARE, view order

## Abstract

**Purpose:**

To provide explicit, vendor‐independent view‐order constructions that enable selection of an arbitrary center echo in multi‐shot imaging, with a focus on RARE and MPRAGE.

**Methods:**

Vendor‐independent view‐order methods were developed to assign echo and shot indices to a prescribed set of phase encodes, enabling explicit selection of the center echo and avoiding large discontinuities near k‐space center. The view‐order methods were implemented in 2D RARE, 3D RARE, and MPRAGE, and evaluated in phantom and in vivo experiments, where the effects of the different methods were explored. An interactive dashboard was provided to reproduce them.

**Results:**

All methods enabled placing the chosen echo/shot index at the k‐space center without changing other scan parameters. In RARE, varying the center echo produced the expected shift in $T_{2}$ weighting. In MPRAGE, varying c produced the expected shift in $T_{1}$ weighting, in agreement with simulations. Artifact patterns in RARE were consistent with discontinuities and symmetry properties of the echo‐index distribution.

**Conclusion:**

Arbitrary‐center view orders decouple scan parameters such as echo train length, matrix size, and undersampling factors, from center‐echo selection. The presented constructions are compatible with common sampling strategies (e.g., partial Fourier, parallel imaging, and elliptical coverage) and sequences. Arbitrary center echoes allow for more control over image contrast and present an additional degree of freedom in sequence design.

## 1 Introduction

In Cartesian MRI, images are formed by acquiring k‐space lines at multiple phase‐encoding positions. When sequences acquire multiple phase‐encode lines while the signal evolves (e.g., during $T_{1}$ recovery or $T_{2}$ decay), we refer to the bundled acquisition of the signal as an “echo train.”

In RARE (FSE/TSE), an echo train consists of multiple spin echoes following a single excitation, whereas in MPRAGE it consists of multiple excitation pulses and their corresponding gradient echoes following inversion. The signal evolution is therefore mapped onto the acquired phase‐encode lines, producing a nonuniform k‐space weighting.

Any sequence that acquires k‐space in multiple echo trains (or shots) must decide on a shot order, that is, how k‐space is acquired from shot to shot. The shot order can be adjusted for the specific application, such as enhancing arterial and venous image contrast in a contrast‐enhanced angiography sequence [1, 2], or reducing motion artifacts [3].

Similarly, any sequence with more than one phase encode per shot must also decide on the echo order, that is, how to distribute the phase encodes *within* a shot. View order is the combination of shot and echo order, and is an important choice in any sequence design.

Both MPRAGE and RARE are multi‐shot sequences with large echo train lengths (ETL), where view order is of particular importance [4, 5]. In RARE, a single excitation pulse followed by multiple refocusing pulses form the echo train. In MPRAGE, multiple excitation pulses make up the echo train. The echo order in MPRAGE influences image contrast and SNR, while shot order affects motion and post‐contrast delay sensitivity.

View orders for MPRAGE have been proposed not only to improve SNR and contrast [6, 7] but also to support retrospective motion correction applications [8].

In RARE, the echo order determines the $T_{2}$ modulation transfer function (MTF) and the image contrast is almost entirely determined by the phase encodes near the k‐space center. In combination with the refocusing flip angle scheme, the echo order determines the effective echo time ${\text{TE}}_{\text{eff}}$ [9].

Long echo trains are strongly affected by $T_{2}$ signal modulation, resulting in $T_{2}$‐blurred images [10]. A strong intensity difference in k‐space causes image artifacts, hence a distribution where adjacent phase encodes have the same TE is beneficial. We define a segment as the set of phase encodes assigned to the same echo index (and thus the same TE).

**FIGURE 1:**
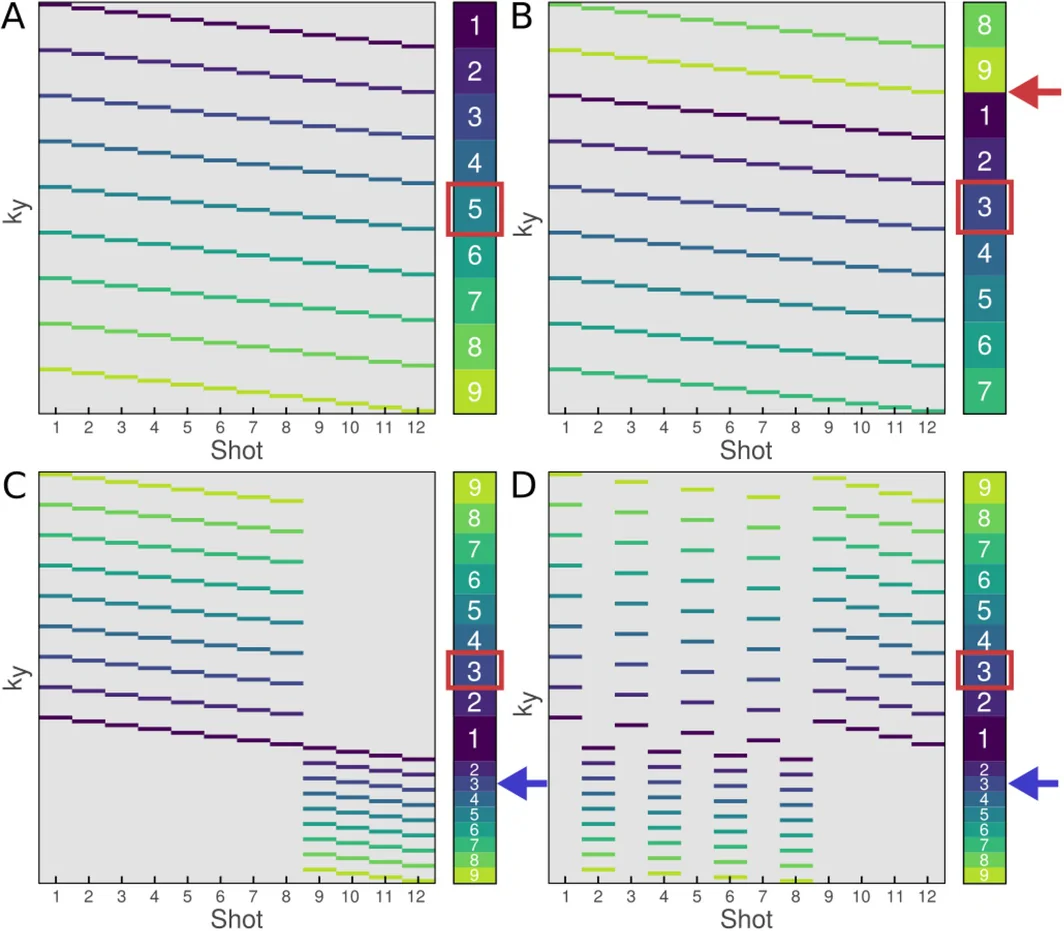
Linear view orders for a 2D RARE sequence with ETL=9 echoes. The subplots to the right of each view order show the projection across shots. **(A)** Sequential echo order, c=5 is determined implicitly. **(B)** Sequential order with circular shift, explicit c=3. A large signal discontinuity is indicated with a red arrow. **(C)** Split‐segment sequential order with c=3. The center segment is smaller since segments are split (blue arrow). **(D)** Same as C, but with interleaved *shot* order.

Even when segmenting phase encodes, there are ETL! available segment arrangements to choose from. It is clear that intensity differences *between* segments remain an issue, which can be reduced by arranging the segments sequentially across k‐space. This constitutes the sequential echo order, where Figure 1A shows a 2D example. However, the center echo, c, is implicitly determined to be ETL/2 in sequential order. Only by modifying the set of phase encodes, for example, partial Fourier, can c be adjusted. For long echo trains, ${\text{TE}}_{\text{eff}}$ with sequential order can be too high for the clinical protocol, hence the need for arbitrary selection of c.

RARE sequences are ubiquitous in clinical MRI, and major vendors allow the user to prescribe ${\text{TE}}_{\text{eff}}$. However, the corresponding view‐order implementations that realize an arbitrary center echo are typically proprietary and have not been described in the public domain.

For MPRAGE, mostly sequential or variants of centric (i.e., c=1) have been described in the literature [11, 12, 13]. Sequential ordering in MPRAGE shares the same limitations as RARE, that is, c=ETL/2. Moreover, in MPRAGE, ETL typically equals the number of phase encodes within a $k_{y}$ plane, such that changes in the number of partitions or in the acceleration factor along $k_{z}$ may also change image contrast. Although methods that lift this ETL constraint have been described in the literature [12], this flexibility is not available from all vendors.

In existing implementations, the center echo is therefore either implicitly determined by the choice of view order, or controlled through vendor‐specific and unpublished methods. As a result, there is no general, vendor‐independent framework for selecting the center echo independently of other scan parameters.

We describe several view‐order techniques that enable an arbitrary center echo and are compatible with any prescribed set of phase encodes, including Poisson disc sampling [14], partial Fourier, 2D‐CAIPIRINHA [15], and conventional parallel imaging sampling patterns [16, 17]. The differences between view ordering in RARE and MPRAGE are analyzed and demonstrated experimentally.

## 2 Methods

View ordering is defined with respect to a prescribed set of phase encodes, that is, $k_{y}$ or ($k_{y},k_{z}$) coordinates. This set is determined by the desired matrix size together with any acceleration strategy, such as parallel imaging, partial Fourier, or elliptical 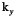
–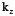 coverage. With elliptical 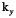
–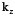 coverage, the prescribed phase encode set is obtained by truncating the Cartesian 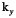
–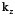 grid to its inscribed ellipse [9, 12, 18].

Once the phase encode set is fixed, the remaining problem is to decide when each phase encode is acquired. Specifically, out of $N_{k}$ phase encodes, each must be assigned both an echo index within the echo train and a shot index within the acquisition. In the terminology used here, the echo assignment determines the echo order (and thus signal weighting), whereas the shot assignment determines the shot order (and thus the temporal ordering of k‐space sampling). A view order provides an explicit construction for these assignments. Depending on the method, it may also allow explicit selection of the center echo c.

Given $N_{k}$, each phase encode must be assigned an echo between 1 and ETL. Since there are

(1) $$N_{\text{shots}}=\left\lceil \frac{N_{k}}{\text{ETL}}\right\rceil$$

shots necessary to acquire all phase encodes, each echo index will be used up to $N_{\text{shots}}$ times across the acquisition. As implied by Equation (1), any acceleration reduces the number of phase encodes available per segment, which can affect the MTF. Note that from the ceiling operator in Equation (1), there will be $N_{\text{shots}}\times \text{ETL}$ echoes, which typically exceeds $N_{k}$. These superfluous echoes are usually discarded and are not further considered here.

A general view order method should satisfy four structural requirements. First, it should avoid large discontinuities in echo index across k‐space, particularly near the center. Both echo and shot order are relevant here, since echo order modulates signal weighting while shot order influences motion sensitivity. Second, the construction should be compatible with any prescribed set of phase encodes. Third, it should allow explicit selection of c. Finally, it should be defined independently of specific scan parameter values, such that changing ETL or matrix size does not alter the fundamental construction.

In practice, view‐order design follows four steps: choosing a reference point in k‐space ($k_{0}$), partitioning k‐space into segments, assigning echoes to segments (linearly or via a permutation), and assigning a shot order.

Below, we first present the naive approach for achieving an arbitrary center echo (circular shift). We then present four increasingly complex view ordering methods derived from the steps above and satisfying the stated requirements. Finally, we demonstrate how view‐order choice affects image appearance in RARE and MPRAGE.

### 2.1 Sequential Order With Circular Shift

The simplest way to extend sequential order to an arbitrary center echo, c, is by circularly shifting segments, similar to bit rotation. However, a large discontinuity appears at the interface of the first and last echo segment, which can cause ghosting, indicated with a red arrow in Figure 1B. This approach was first described in the context of a 2D inversion‐prepared spoiled gradient echo sequence, where altering echo order relative to the inversion pulse modulated $T_{1}$ weighting [19].

If there are multiple shots, this discontinuity can be avoided by splitting segments. The fraction to split is determined by c. Each set of shots can then be acquired either sequentially (Figure 1C) or interleaved (Figure 1D). In the former case, the first part of the scan is equivalent to a sequential order with partial Fourier along the phase encoding direction, while the end of the scan fills in the missing region. In the latter case, the initial pair of shots yields a sparse k‐space coverage with full extent, equivalent to an accelerated acquisition, and the missing data are filled in by later shots. Note that both cases will result in the same echo order (i.e., same MTF), marking the difference between echo order and shot order. While splitting allows for an arbitrary center echo, it also creates smaller segments, which negatively impacts the MTF.

Splitting segments is inefficient when the number of phase encodes within either set of shots, divided by ETL, deviates from an integer value, leading to superfluous echoes that cannot be assigned a phase encode. Further, a fraction of the most desirable echoes (those closest to the selected c) will be situated at high spatial frequencies in k‐space rather than the center. This split is indicated by the blue arrow in Figure 1C,D.

### 2.2 Linear Center Pivot Outer‐LCPO

Instead of splitting, segments can be permuted to achieve the desired c. LCPO acquires k‐space center linearly (LC), minimizing discontinuities.

The number of linear center segments, $N_{\text{lc}}$, depends on c:

(2) $$N_{\text{lc}}=\left\{\begin{array}{ll} 2c-\text{ETL}\;\mathrm{mod}\;2,\; & c\leq \text{ETL}/2\\ 2\left(\text{ETL}-c\right)+\text{ETL}\;\mathrm{mod}\;2,\; & c>\text{ETL}/2 \end{array}\right.$$

Remaining outer segments ($N_{\text{po}}=\text{ETL}-N_{\text{lc}}$) are assigned in a pivoting fashion (PO, pivot outer), alternating to distribute the echoes more evenly compared to circular shift, forming the LCPO order.

**FIGURE 2:**
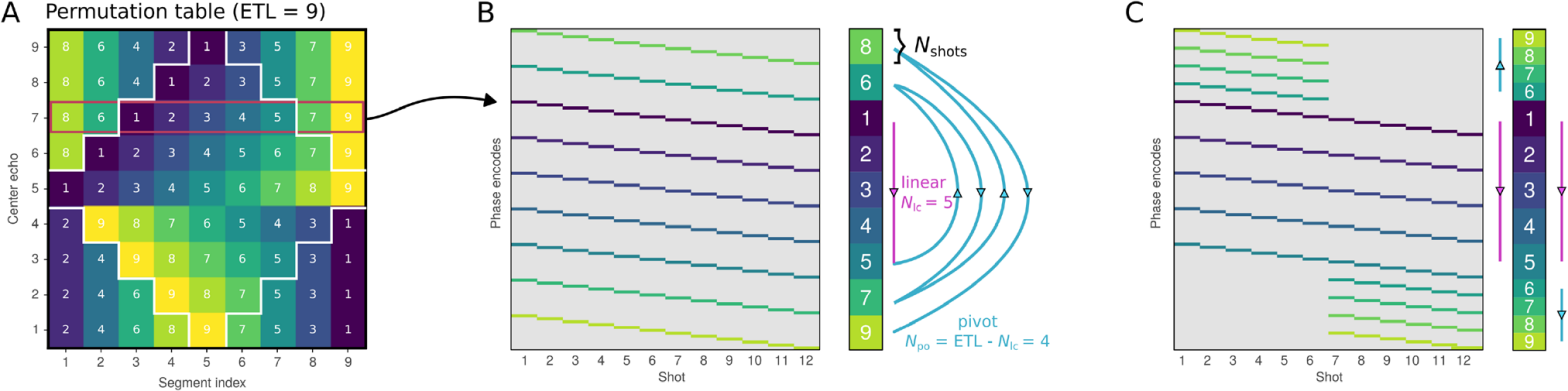
LCPO view order with ETL=9. **(A)** Permutation table where each row shows the permutation for a specific center echo, according to Equation (2). The white region marks the linear section. **(B)** LCPO view order with c=3 with 12 shots. **(C)** Same as B, but with split segments.

If the phase‐encode density is symmetric in the phase encode direction, segments can be assigned linearly along $k_{y}$ (or $k_{z}$ in case of 3D imaging). If asymmetric (e.g., partial Fourier), LCPO order can be achieved using a sorting algorithm with two parts, LC and PO:

1. **LC**: Sort all $N_{k}$ phase encodes by their distance from the k‐space center, $k_{r}$.
2. Select the subset of $N_{\text{shots}}\times N_{\text{lc}}$ phase encodes with the smallest $k_{r}$.
3. Sort the subset by $k_{y}$ (or $k_{z}$) and set $k_{0}$ to the encode with the smallest $k_{y}$/$k_{z}$.
4. Create $N_{\text{lc}}$ partitions, each containing $N_{\text{shots}}$ phase encodes.
5. Assign echoes to each segment linearly.
6. **PO**: Now there are $N_{k}-N_{\text{shots}}\times N_{\text{lc}}$ unassigned encodes. Choose one of the following:
Split segments:
Sort remaining phase encodes by $k_{r}$ and partition into $2\times (\text{ETL}-N_{\text{lc}})$ segments. Then assign echoes to segments in pairs according to their $k_{r}$ distance (Figure 2C).
Whole segments:
Sort remaining phase encodes by $k_{y}$ (or $k_{z}$) and partition into $\text{ETL}-N_{\text{lc}}$ segments, then assign echoes in a pivoting fashion (Figure 2B), which permutes the order.
7. Assign the shot order, either interleaved or sequentially, as described in Section 2.1.

If c>ETL/2, segments are instead assigned decreasingly, starting from ETL. An example of LCPO with ETL=9 is shown in Figure 2 with all possible center echoes in a permutation table. In a multi‐shot acquisition, the outer segments can be split for a smoother MTF, as shown in Figure 2C.

### 2.3 Pivot Center Linear Outer‐PCLO

With the additional direction $k_{z}$ in 3D comes the ability for view orders in auxiliary directions to the primary phase encoding axes, such as radially in the 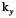
–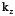 plane. In this $k_{r}$ case, rings rather than rows or columns define the segments. Radial echo order is beneficial because the point spread function (PSF) becomes more isotropic, which can improve reformats [9]. Combining radial MTF with linear echo order starting from the innermost ring (i.e., centric) with azimuthal shot order achieves a short TE sequence where each shot acquires a spoke in the 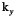
–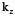 plane [9, 20]. In this case, c is always 1.

With radial segments, it is not possible to apply LCPO or split segments, since rings cannot have negative radii.

Instead, an arbitrary center echo can be achieved by permuting $N_{\text{PC}}=c+(c\;\text{mod}\;2)$ central rings and acquiring the outer rings sequentially, described by the permutation:

(3) $$P_{\text{PCLO}}(i,c)=\left\{\begin{array}{l} \begin{array}{cccc} c-2i+2, & & & i\leq \frac{N_{\text{PC}}}{2}\\ 2i-c-1, & & \frac{N_{\text{PC}}}{2}< & i\leq N_{\text{PC}}\\ i, & & & i>N_{\text{PC}} \end{array} \end{array}\right.,$$

where i∈[1,ETL] is the echo index. Equation (3) is valid for c≤ETL/2. For larger c, the permutation is simply $\text{ETL}+1-P_{\text{PCLO}}(\text{ETL}-c+1)$.

**FIGURE 3:**
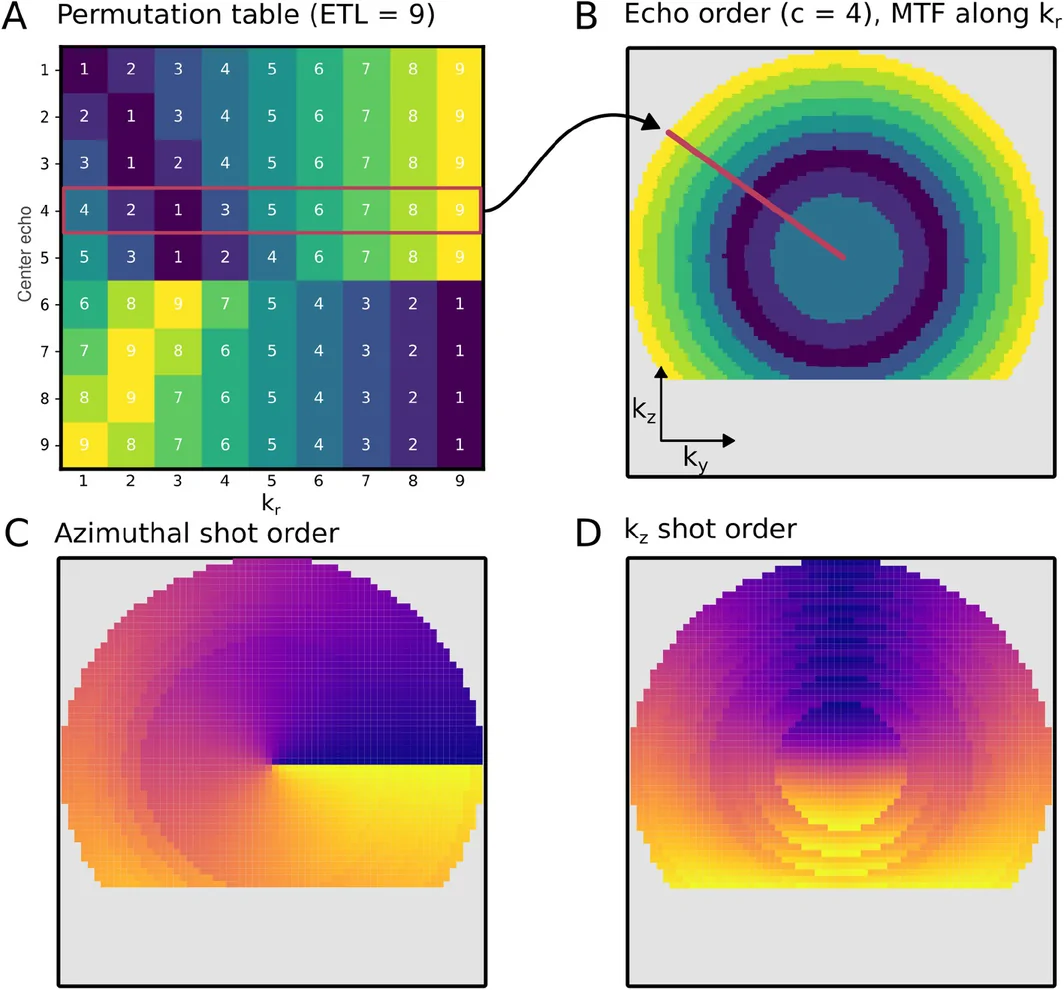
PCLO view order for a 3D RARE sequence with ETL=9 radial echo segments and a partial Fourier acquisition with elliptical k‐space coverage. **(A)** Permutations for all possible center echoes with PCLO, calculated from 3. **(B)** The echo order for c=4. **(C)** Azimuthal shot order. **(D)** Shot order along 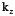. Early shots are colored blue and later shots are yellow.

The PCLO algorithm proceeds as follows:

1. Set $k_{0}$ to the k‐space origin.
2. Sort phase encodes by their radial distance from $k_{0}$ and partition into ETL segments.
3. Assign echoes to each phase encode from the permutation described by Eq. 3.
4. Assign a shot order, for example, azimuthal (Figure 3C) or linear along $k_{y}$ (Figure 3D).

The resulting view order acquires central rings by pivoting (PC, pivot center) until the innermost segment has been acquired. The following outer segments are acquired linearly (LO, linear outer). PCLO is a generalization of the centric view order, with all permutations for ETL=9 shown in Figure 3A. Also shown is the echo order for c=4 together with two different shot orders (C–D).

It should be noted that both LCPO and PCLO can be used in both 2D and 3D imaging, with the modification that PCLO permutes $k_{y}$ column segments instead of $k_{r}$ rings.

### 2.4 A Note on Shot Order

Shot ordering is a separate design problem from echo ordering. Any given echo order (e.g., Figure 3B) can be paired with different shot orders. For example, shots can traverse segments by increasing azimuthal angle in the 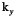
–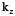 plane (Figure 3C), or they can be assigned by stepping through segments in order of $k_{y}$ or $k_{z}$ (Figure 3D). The azimuthal strategy introduces a sampling singularity near the k‐space center, whereas the $k_{y}$‐ordered strategy avoids it.

In 3D acquisitions, the phase encodes lie in a two‐dimensional k‐space plane (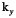
–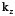), so shot order cannot be visualized as straightforwardly as in Figures 1 and 2. Instead, we pseudo‐color shot and echo order with different colormaps to distinguish them.

Single‐shot acquisitions represent the limiting case in which all prescribed phase encodes are acquired within a single readout train, and the shot order becomes trivial. Circular shift, LCPO, and PCLO (along $k_{y}$) are compatible with single‐shot acquisitions, including RARE sequences such as HASTE as well as PROPELLER, where each blade is acquired in a single shot [21]. Chevron and CROC are multi‐shot methods and described in the next subsections.

### 2.5 Chevron

**FIGURE 4:**
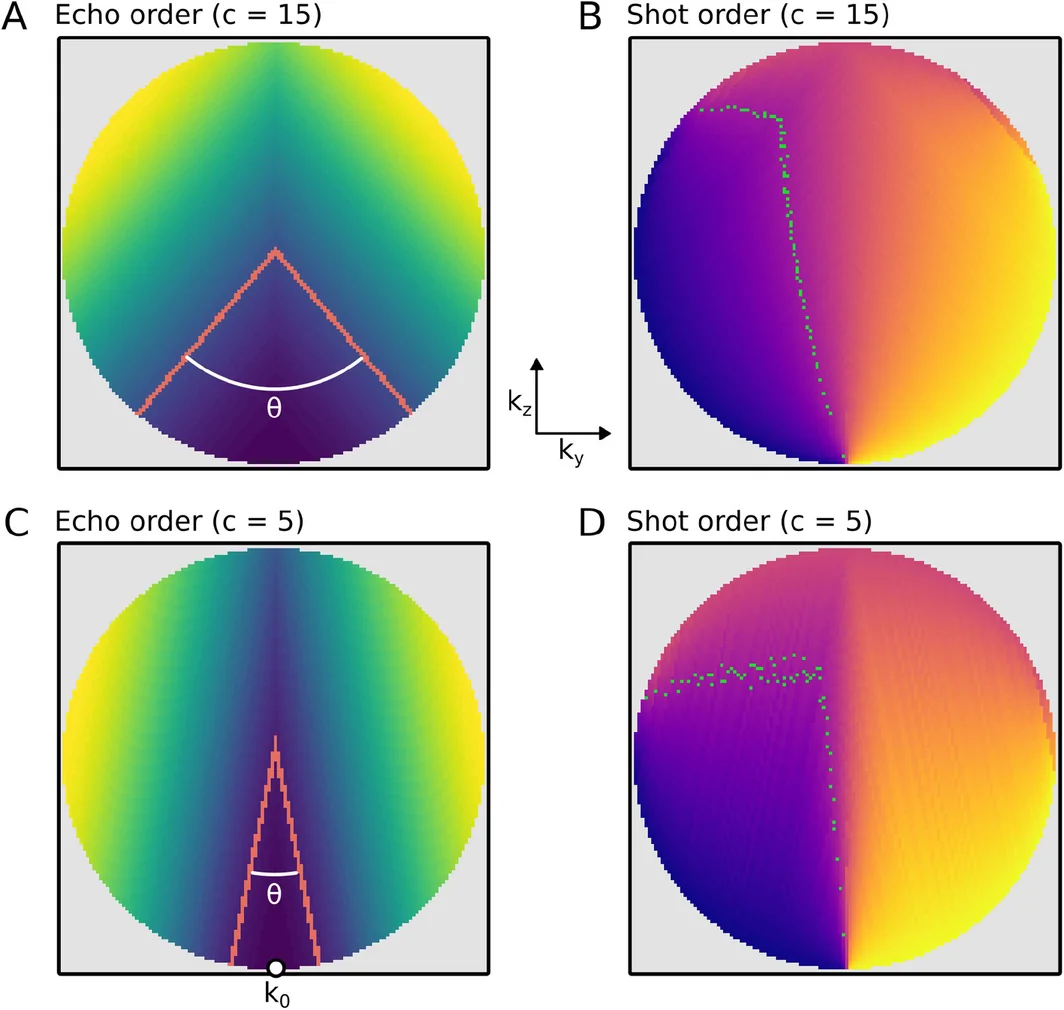
Chevron view order for a 3D RARE sequence with **(A)** center echo = 15, **(C)** center echo = 5. **(B)** and **(D)** Azimuthal shot order around ${\text{k}}_{0}$, with a shot highlighted in green. Early shots/echoes are colored blue, late are yellow. Increasing the internal chevron angle θ yields a larger c.

Further building on auxiliary coordinates in 3D imaging, another view order was designed to distribute echo indices smoothly and approximately uniformly over the 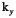
–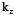 plane. Rather than assigning segments as straight rows/columns or concentric rings, this method assigns segments along V‐like bends, hence referred to as Chevron. The echoes are assigned by distance from an origin $k_{0}$, chosen as a peripheral phase encode in the 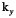
–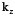 plane as shown in Figure 4A. The choice of internal chevron angle θ determines which segment intersects k‐space center, thus assigning the center echo index (Figure 4A vs. C).

In the case where the phase‐encode density is radially symmetric, segments [1,c] are in the sector spanned by θ (Figure 4A), leading to a simple analytical solution: θ=2πc/ETL. In the general case, θ can be found algorithmically as follows:

1. Initialize the internal chevron angle θ=0.
2. Choose a reference point on a k‐space edge, for example, $k_{0}=\left(y_{\max},\frac{z_{\max}}{2}\right)$.
3. For each phase encode $k_{i}=(y_{i},z_{i})$, compute the $\ell_{1}$‐distance from $k_{0}$:
(4) $$d_{i}=\left|y_{i}-y_{0}\right|+\left|z_{i}-z_{0}\right|\cdot \tan\left(\frac{\theta }{2}\right)$$
  Here, the tangent function stretches/squeezes the chevron pattern.
4. Partition k‐space into ETL chevron segments by sorting the phase encodes by d
5. Assign echoes linearly from 1 to ETL to each segment.
6. Retrieve current c from the assigned echo in the phase encode closest to the k‐space center.
7. Increase θ and repeat steps 3–6 until the desired center echo is obtained.
8. Assign an azimuthal shot order from $k_{0}$.

Since increasing θ monotonically increases c, a suitable value of θ can be efficiently found using binary search.

Figure 4 illustrates the Chevron view order for two different choices of center echo. Panels B and D also show the corresponding azimuthal shot order around $k_{0}$, with a highlighted shot almost halfway through the scan.

### 2.6 Concentric Rings With Offset Center‐CROC

**FIGURE 5:**
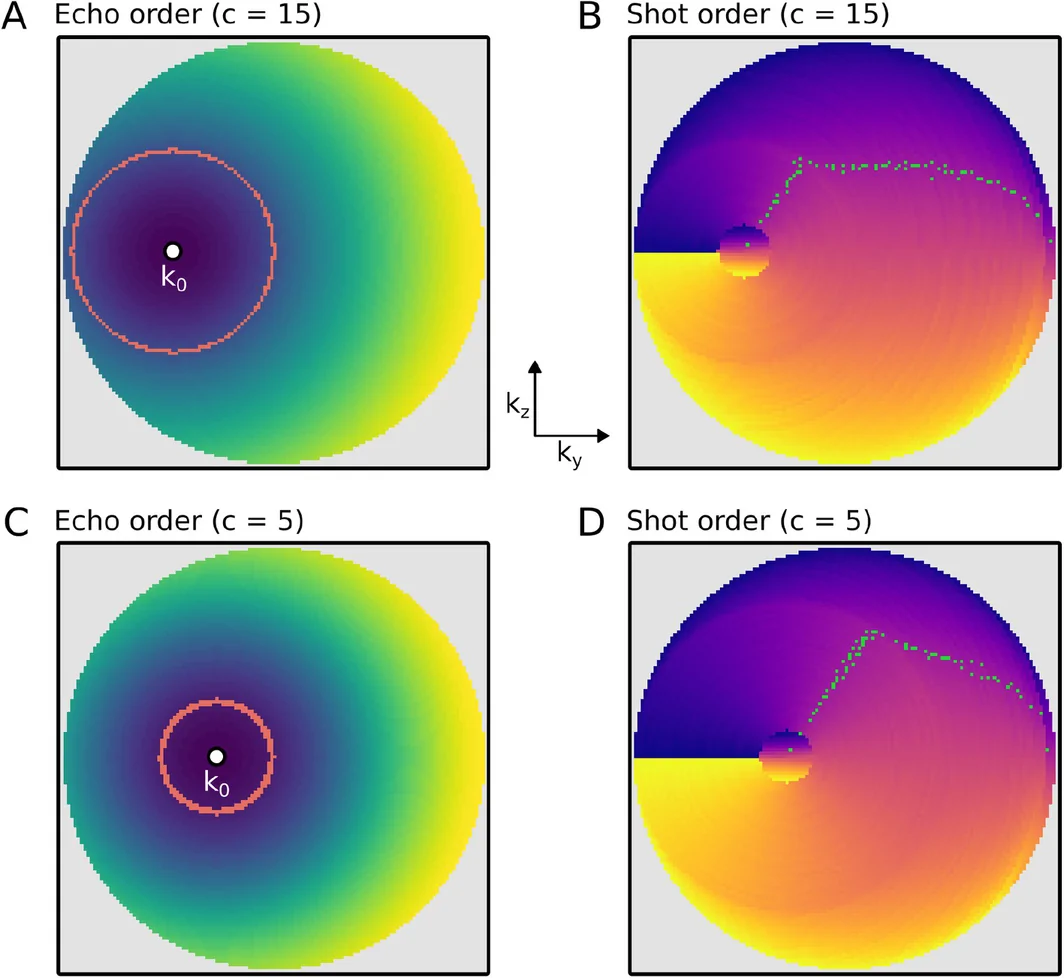
CROC view order for a 3D RARE sequence with **(A)** center echo = 15, **(C)** center echo = 5. **(B)** and **(D)** Azimuthal shot order around ${\text{k}}_{0}$, with a shot highlighted in green. Early shots/echoes are colored blue, late are yellow. c is increased when ${\text{k}}_{0}$ is farther away from the k‐space origin.

Similar to Chevron, another view order was designed to distribute echo indices smoothly and approximately uniformly over the $k_{y}$–$k_{z}$ plane. In addition, this method aims to improve the circular symmetry of the echo order, which can be desirable when reformat quality is prioritized. PCLO achieves an arbitrary center echo by permuting a set of central rings. In contrast, this method offsets the reference point $k_{0}$ away from the k‐space origin. This changes which ring is assigned to the k‐space center and thereby increases c, as illustrated in Figure 5. We refer to this method as CROC as it forms an echo order with concentric rings with an offset center. If the desired c exceeds the maximum achievable by placing $k_{0}$ at the edge of the prescribed k‐space, a higher c can be obtained by distributing echoes along concentric ellipses rather than circles. The algorithm proceeds as follows:

1. Identify the densest direction from the k‐space center toward the periphery and place the initial reference point at the corresponding edge, for example, $k_{0}=\left(\frac{y_{\max}}{2},z_{\max}\right)$.
2. For each phase encode $k_{i}$, compute the elliptical distance from $k_{0}$:
(5) $$d(k_{i},k_{0})=\sqrt{{\left(y_{i}-y_{0}\right)}^{2}+\frac{{\left(z_{i}-z_{0}\right)}^{2}}{q^{2}}}$$
  where q determines the elliptical aspect ratio (initially 1).
3. Partition k‐space into ETL elliptical segments by sorting the phase encodes by d.
4. Assign echoes linearly from 1 to ETL to each segment.
5. Retrieve the current center echo c from the assigned echo in the phase encode closest to the k‐space center.
6. If the desired center index is smaller than c, shift $k_{0}$ toward the k‐space center; if it is larger, adjust q.
7. Repeat steps 2–6, updating either q or $k_{0}$ via binary search until the desired c is at the k‐space origin.
8. Assign an azimuthal shot order from $k_{0}$. To avoid a singular point from which shots from the whole scan originate, the first segment is assigned linearly along $k_{z}$ or $k_{y}$.

The final step highlights an additional degree of freedom in view order methods: For a given segment, the order in which its phase encodes are distributed across shots can be chosen independently. Here, by replacing the first segment with a linear order rather than azimuthal, the singularity in Figure 3C is avoided. This effect can be seen in Figure 5B and D. Figure 5A and C additionally show how $k_{0}$ is displaced as c is increased.

### 2.7 Interactive View Order and MPRAGE Bloch Simulator

We developed an open‐source interactive web‐based dashboard that calculates the proposed view orders and allows real‐time adjustment of parameters such as ETL, c, matrix size, partial Fourier, parallel imaging with CAIPIRINHA, and supports elliptical $k_{y}$–$k_{z}$ coverage.

The dashboard is intended to build intuition for how scan parameters affect the view order. It also includes an MPRAGE Bloch simulator that displays tissue signals across the echo train, with adjustable scan parameters.

In addition, to support reproducible use in external implementations, the dashboard can export a serialized description (JSON) of the prescribed sampling pattern and view order, which can be used in open‐source pulse sequence frameworks such as Pulseq [22], mtrk [23], and gammaSTAR [24]. It is available at https://henricryden.github.io/vieworder/.

### 2.8 Experiments

The view order methods were implemented in in‐house‐developed RARE and MPRAGE sequences using the KS Foundation framework [25]. View orders were calculated directly on the host computer with negligible calculation times.

All images were acquired on a 3T Signa Premier (GE Healthcare). Images were reconstructed by the vendor reconstruction, without denoising. Four in vivo experiments were performed, where informed consent was obtained from all volunteers in accordance with the institutional review board policy.

#### 2.8.1 RARE

Refocusing flip angles were designed using the EPG‐based pseudo‐steady‐state signal envelope method [9]. The same flip angles were used regardless of view‐ordering scheme and were obtained from the vendor‐provided protocol.

High resolution sagittal 2D‐RARE images with musculoskeletal PD weighting were acquired with ${\text{TE}}_{\text{eff}}$ = 30 ms of the ankle with a dedicated 16‐channel receive coil. Circularly shifted view order was compared with LCPO. Relevant scan parameters were TR = 3 s, ETL = 16, c=4, matrix size 512×512, FOV = 240×240 mm.

Second, a 3D RARE $T_{2}$‐FLAIR vendor‐provided brain protocol with a 48‐channel head coil was used to evaluate the effect of view order. Relevant scan parameters were TR = 6.3 s, ETL = 220, scan time 6:31, ${\text{TE}}_{\text{eff}}$ = 120 ms, elliptical $k_{y}$–$k_{z}$ coverage, matrix size 256×242×120, FOV = 256×242×120 mm. Sequential view order required ETL = 150 to match ${\text{TE}}_{\text{eff}}$ (i.e., c=75) and increased scan time to 10:05. Restoring ETL to 220 recovered the original scan time but raised c and ${\text{TE}}_{\text{eff}}$. We applied the proposed view orders to counteract the contrast change and restore the target ${\text{TE}}_{\text{eff}}$ without increasing scan time.

Third, a vendor‐provided $T_{2}$‐weighted cervical spine 3D RARE protocol was used to measure the MTF on a phantom containing compartments representing grey matter and cerebrospinal fluid (CSF). Images were acquired with a 16‐channel head/neck coil. The pulse sequence was modified to remove both phase encoding and readout gradients so that the acquired signal reflects only temporal decay, not modulated by spatial frequency content of the object. For these measurements we turned off parallel imaging acceleration to improve visualization of the MTF. To assess the impact of the view order on the resulting images, the same protocol was repeated *with* imaging gradients on the phantom and in vivo, using standard accelerated settings (R=2) in the in vivo case. Relevant scan parameters were TR = 2 s, ETL = 100, elliptical $k_{y}$–$k_{z}$ coverage, matrix size 244×342×140, FOV = 230×280×70 mm.

#### 2.8.2 MPRAGE

To demonstrate that arbitrary center‐echo assignment can also modulate $T_{1}$ weighting, we applied the proposed view orders to MPRAGE. Sagittal images were acquired with the preparation delay (i.e., time from inversion to *start* of the MPRAGE train) set to 400 ms. 200 echoes were acquired with an echo spacing of 6.7 ms, resulting in an MPRAGE train duration of 1.34 s, followed by a recovery duration of 900 ms. The excitation flip angle was $8^{\circ }$. Matrix size 256×256×140, FOV = 256×256×140 mm, elliptical 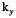
–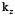 coverage, R=2. Scan time was 3:06.

To show the compatibility with partial Fourier, we also acquired MPRAGE data on a phantom with the same protocol, but with truncated phase encodes along $k_{y}$ and R=1. A partial Fourier reduction factor of 0.7 was used, resulting in a scan time of 3:56. Fully sampled data was acquired for reference, acquired in 5:37.

Images were acquired with a 48‐channel head coil. The proposed view order methods were applied with c=67 and c=133. The scanned protocol was simulated with the MPRAGE Bloch simulator. A sequential reference was also acquired with c=100.

## 3 Results

**FIGURE 6:**
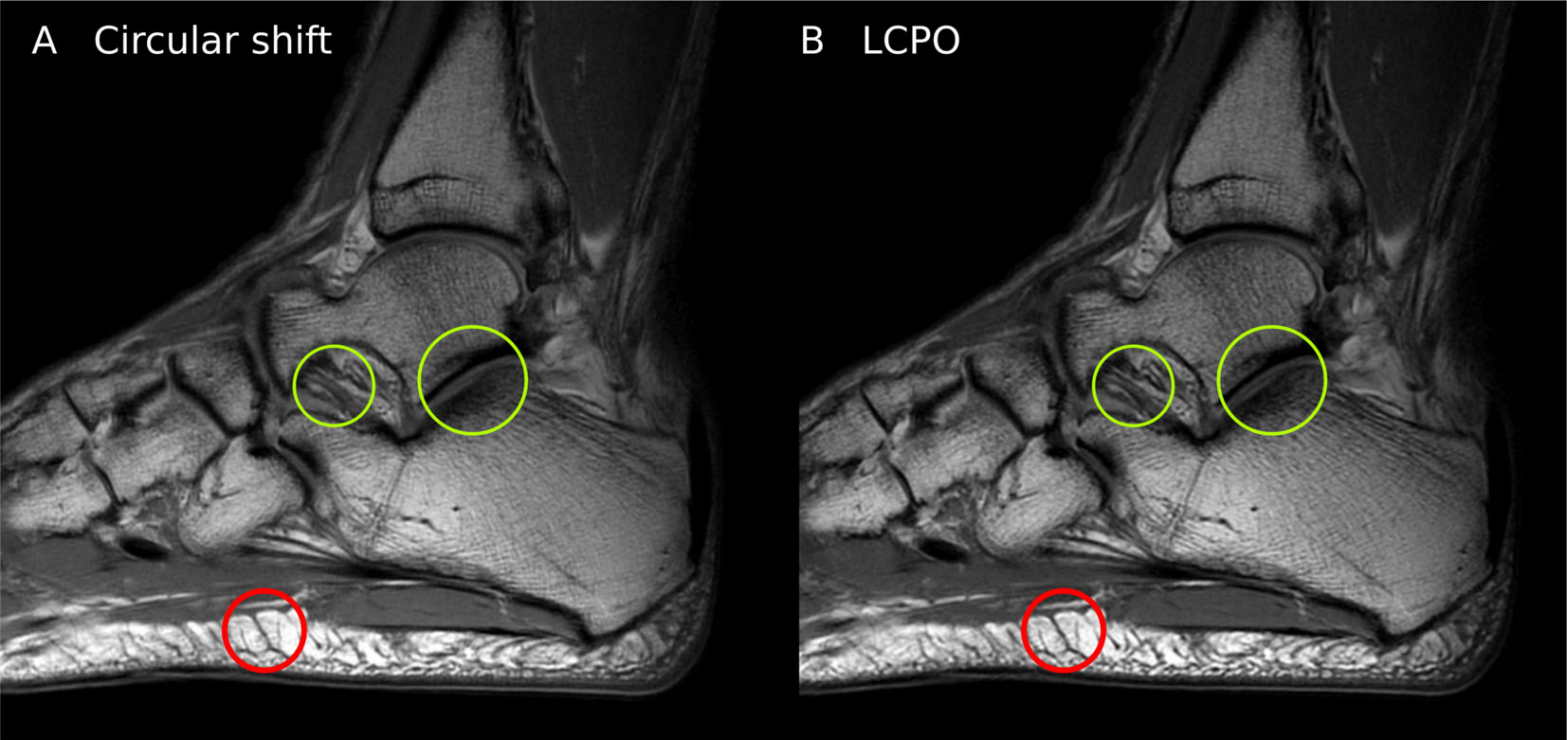
2D RARE sequence with MSK PD weighting (${\text{TE}}_{\text{eff}}$ = 30 ms) and different view orders. ETL was 16 and the center echo was c=4. With LCPO, there is better delineation of the thin cartilage in the talocalcaneal joint and of the ligament (green circles), bone trabeculae, and subcutaneous tissue (red circle).

Figure 6 shows that image sharpness is improved with LCPO over circularly shifted segments. The improvement in sharpness is particularly visible in the trabecular structures.

**FIGURE 7:**
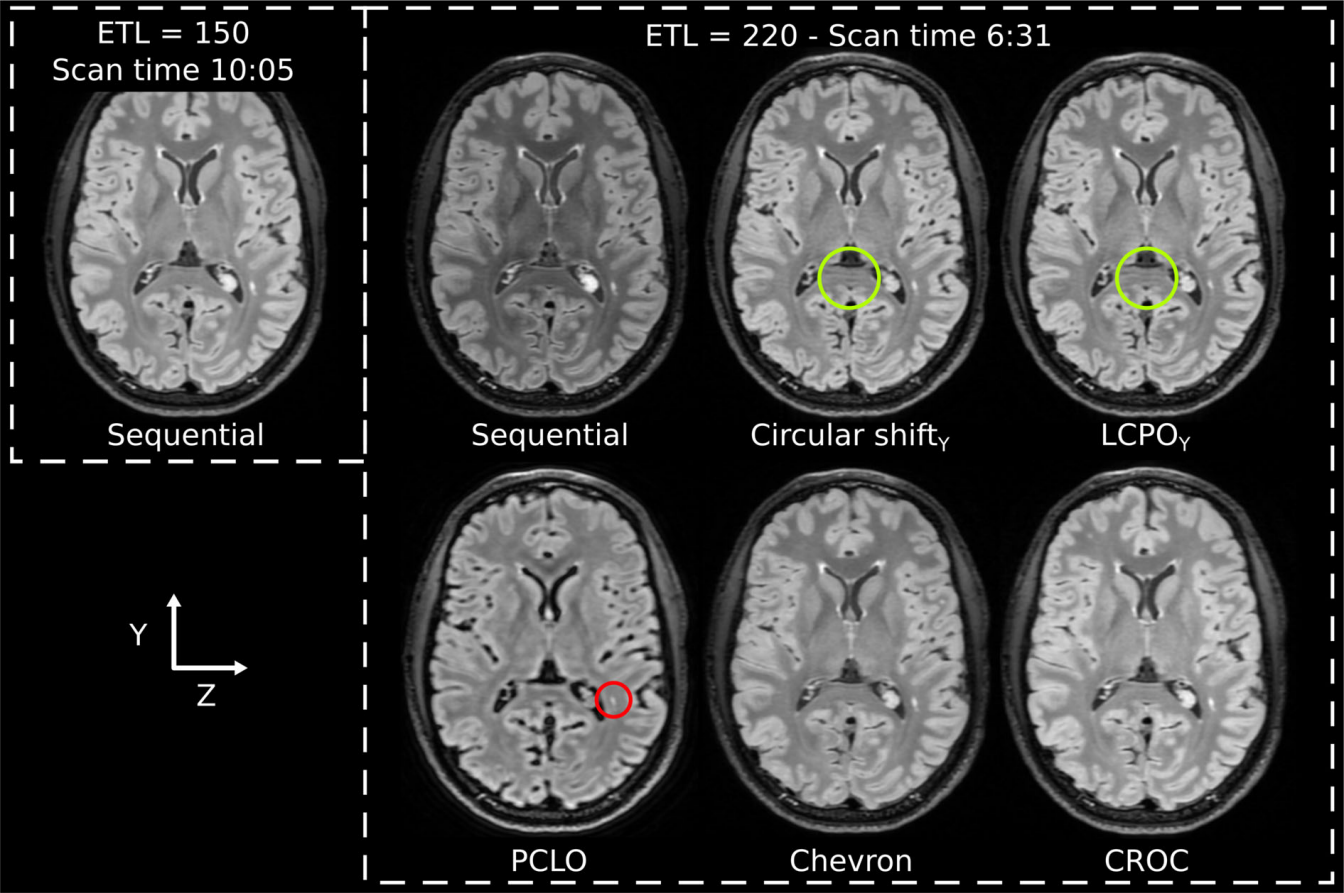
3D T2 FLAIR RARE acquired with vendor‐provided default parameters (ETL = 220, ${\text{TE}}_{\text{eff}}$ = 124 ms). An ETL of 220 yields images with an undesired ${\text{TE}}_{\text{eff}}=174$ ms. For sequential, ETL was decreased from 220 to 150 to match the desired ${\text{TE}}_{\text{eff}}$, resulting in a scan time of 10:05. The proposed view orders were acquired with a center echo chosen to match ${\text{TE}}_{\text{eff}}$ = 124 ms, with a scan time of 6:31. Green circles highlight a ringing appearance along Y. The red circles highlight a partly obfuscated white matter lesion.

All five view orders with arbitrary center echoes restored the image contrast in RARE despite the increased ETL, as shown in Figure 7. Circular shift and LCPO have some ringing in the Y direction (green circles), caused by the previously mentioned signal discontinuity. PCLO produces isotropic blurring, partly obfuscating the white matter lesion in the left hemisphere (red circle). Chevron and CROC are both similar to the ETL = 150 image.

**FIGURE 8:**
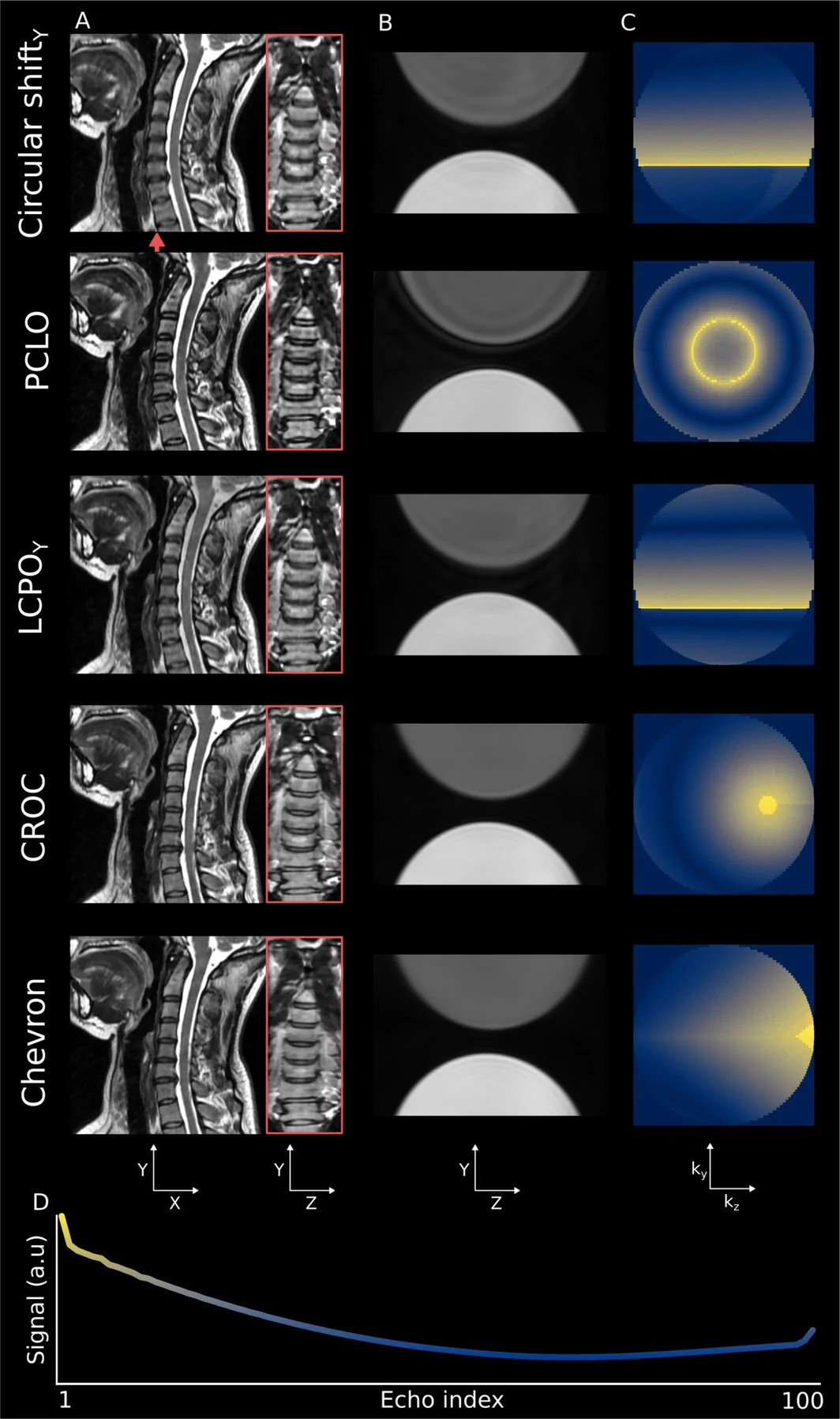
In vivo images and modulation transfer function (MTF) measurements for 3D RARE of a cervical spine protocol for different view orders. **(A)** Sagittal and coronal images for each order acquired in vivo. The red arrow marks the coronal plane. There is a clear difference in vertebral delineation between the different view orders. **(B)** In vitro acquisition of two bottles: the top bottle was filled with a liquid that mimics gray matter in $T_{1}$ and $T_{2}$, and the bottom bottle mimicked CSF. **(C)** MTF measurements (yellow: high signal, blue: low) on a phantom acquired without encoding gradients. **(D)** Signal evolution over the RARE echo train. The view order determines how this measured signal evolution is mapped onto k‐space.

Figure 8C shows the measured k‐space weighting for the corresponding view order, with LCPO and circular shift having a clear signal discontinuity along $k_{y}$. PCLO also has a ring‐shaped signal discontinuity. The MTFs span from high signal (yellow) to low signal (blue) in a nonmonotonic fashion. Panel D shows the RARE echo‐train signal envelope, suggesting that this pattern arises from flip‐angle modulation rather than from the view order.

Both Chevron and CROC avoid discontinuities. The top row shows the images, with a coronal (Y–Z plane) reformat marked in red. A corresponding Y–Z reformat from the phantom experiment is also shown. As expected, view‐order effects are stronger in the gray‐matter mimicking bottle than in the CSF‐mimicking bottle because $T_{2}$ is shorter. There is a clear difference in vertebral delineation between the different view orders, consistent with the ringing artifacts in the phantom scan (B) and the corresponding MTF behavior along $k_{y}$ (C).

**FIGURE 9:**
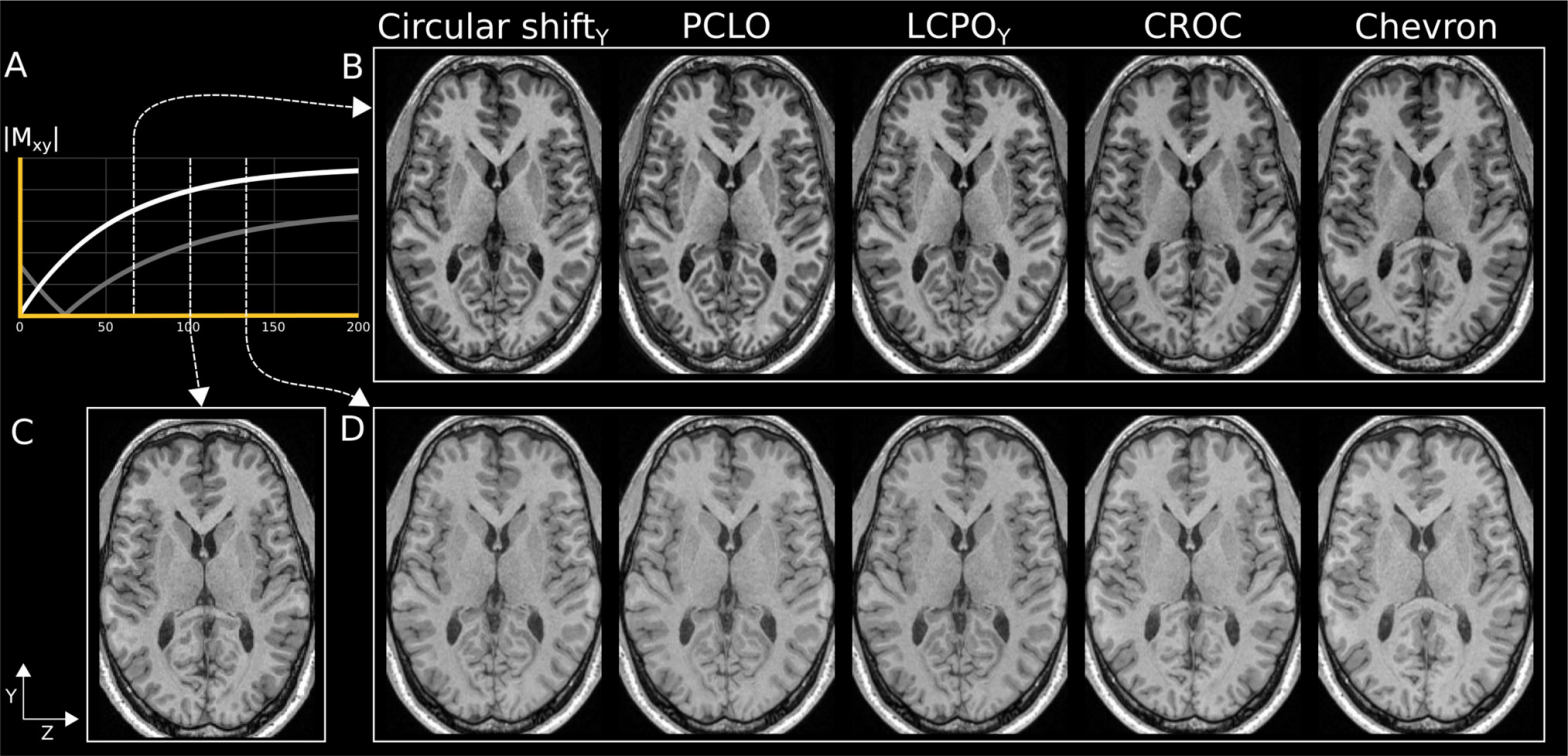
MPRAGE acquisition with different view orders. c was changed to modify $T_{1}$ contrast. **(A)** Bloch simulated transverse magnetization during the MPRAGE train for gray and white matter as a function of echo index. **(B)** In vivo image acquired with c=67, showing increased gray–white matter contrast. **(C)** Reference in vivo image acquired with c=100 and sequential view order. **(D)** In vivo image acquired with c=133, showing decreased contrast.

Figure 9 shows in vivo MPRAGE images acquired with two choices of c. Panel A shows the corresponding contrast evolution predicted by the Bloch simulation, which agreed well with the observed contrast changes. All proposed view‐order methods can modulate $T_{1}$ contrast in an MPRAGE sequence through selection of c.

Relative to the sequential reference (c=100), lower c increased gray–white matter contrast, whereas higher c decreased it. Compared with RARE, the differences between view‐order methods are smaller.

The effects of partial Fourier on the resulting view order and reconstructed images are shown in Supporting Information: Figure S1. The truncation of k‐space causes some image artifacts, but the contrast is maintained for all view orders.

## 4 Discussion

This work demonstrates in both RARE and MPRAGE that the degree of freedom in view order extends to sequences that acquire k‐space over multiple shots and/or with multiple phase encodes per shot. In all cases, the temporal evolution of the signal during the readout train is mapped into k‐space by the view order, and the resulting image contrast and artifacts are largely determined by how the k‐space center is sampled.

For example, in MPRAGE the view order determines which inversion times contribute to low spatial frequencies and therefore strongly influences the achieved $T_{1}$ weighting. More generally, whenever the signal evolves during acquisition ($T_{1}$ recovery, $T_{2}$ decay, magnetization transfer, flow, motion), the view order converts that temporal evolution into a spatial‐frequency weighting.

The resulting MTF is application specific. In RARE, the MTF can be calculated from the echo order (integers, e.g., Figure 5A) and the $T_{2}$ decay. In the simplest case with $180^{\circ }$ refocusing pulses and a single tissue, this reduces to an exponential decay. In MPRAGE, the echo order must be considered together with the longitudinal and transverse magnetization states for each echo. Since both MTF formulations depend strongly on tissue and scan parameters, we display echo order in index space to avoid application‐specific bias. For the same reason, and because ROI‐based scalar summaries depend strongly on anatomy, coil sensitivity, filtering, and ROI placement, we prefer the display of k‐space weighting, phantom and in‐vivo image appearance rather than standalone SNR/CNR or artifact‐power metrics in this work. Such scalar metrics may still be useful in task‐specific follow‐up studies such as optimizing visualization of contrast agent uptake.

The point spread function (PSF) is the Fourier transform of the MTF and provides an intuitive view of blurring as a convolution kernel. Its appearance depends on tissue and scan parameters (in RARE: $T_{1}$, $T_{2}$, flip angles, echo spacing), and because it is complex‐valued, it reflects both magnitude and phase. Any phase modulation from echo‐order asymmetry is not captured by the magnitude PSF. By contrast, the echo order shows the ordering of signal weighting across k‐space, and any asymmetry reveals phase modulation in the image domain. For example, PCLO is symmetric (Figures 8 and 3B), indicating no phase modulation while still showing the blurring behavior.

Because vendor view orders are typically unpublished, especially for RARE, view ordering is a vendor‐confounded factor.

Two protocols may nominally match in TR, echo spacing, ETL, and prescribed ${\text{TE}}_{\text{eff}}$, yet differ in the mapping between echo/shot index and phase encodes. Such differences can affect the MTF, image contrast, and artifact behavior (e.g., motion [26]) even when other parameters are identical. This leaves open‐source pulse sequence frameworks without a published, standardized view‐order method, limiting reproducibility. Here we present four such options and provide both the algorithms for generating the view orderings and a dashboard for directly exporting them for use in vendor‐agnostic sequences.

Most clinical MPRAGE sequences use either centric (c=1) or sequential echo order. Centric ordering increases $T_{1}$ weighting by sampling the k‐space center earlier in the signal evolution, but this also increases sensitivity to transient‐state effects and RF inhomogeneity because the signal is farther from steady state at the start of the train [13]. Arbitrary center echoes replace this binary choice with a continuous trade‐off, allowing c to be chosen to balance contrast against robustness.

The MPRAGE experiments show that center‐echo assignment can be used to optimize image contrast within a fixed protocol. The optimal c depends on the desired contrast and can be chosen by maximizing a task‐specific metric (e.g., gray–white matter contrast) based on the signal evolution across the echo train. In practice, c must be considered together with other contrast‐influencing parameters such as the preparation delay, excitation flip angle, ETL, and recovery time. The close agreement between the simulated and measured MPRAGE contrast suggests that simulation can be used as an aid for exploring this parameter space.

As shown in Figure 8C, Chevron places the lowest signal at the highest spatial frequencies, whereas CROC produces a crescent‐shaped fluctuation in the MTF. Similarly, PCLO distributes the lowest signal in a ring with increasing signal further outward. The circular‐shift approach produces the strongest discontinuities, as already described in the Methods section, and it also shows the most prominent artifacts in the images. Taken together, these observations support the conclusion that simple circular shift is inferior to LCPO. In 3D, Chevron and CROC appear to reduce discontinuities more effectively than LCPO, PCLO, and circular shift, although other effects such as gradient fidelity or Eddy currents may also contribute to the observed image differences in Figures 7 and 8.

The purpose of this work is to provide explicit constructions for arbitrary center‐echo assignment. In RARE, the effect of view order is coupled to the refocusing flip‐angle schedule, since the signal envelope along the echo train is mapped into k‐space by the echo order. Besides controlling image contrast in both RARE and MPRAGE, the results show that view order also affects image artifacts and sharpness, particularly in RARE.

The pseudo‐steady‐state signal envelope approach used to design the refocusing flip angles includes control points for the echo assigned to the k‐space center. However, this feature was not used in the vendor implementation, and we likewise did not re‐optimize the flip angles for c. This isolates the effect of view order, such that the observed differences reflect only how a fixed signal envelope is distributed in k‐space. While arbitrary center‐echo assignment does not require optimization of the flip angles, re‐optimization may improve alignment between the signal envelope and c.

Further optimization would need to consider additional scan parameters jointly, together with gradient fidelity and associated Eddy current corrections.

In RARE, this includes refocusing flip‐angle modulation, together with related constraints such as SAR and sensitivity to flow and motion. In MPRAGE, timing delays could potentially be shortened to reduce scan time, while adjusting the center echo to preserve or improve contrast, although this was not explored here. This opens up several directions for future work on joint optimization of center‐echo assignment with other sequence parameters.

## 5 Conclusion

We present explicit, vendor‐independent constructions for view order with arbitrary center echo assignment. The proposed methods are compatible with any prescribed set of phase encodes and avoid structural discontinuities associated with circular shift approaches. These explicit, vendor‐independent constructions provide a reproducible framework for view order in multi‐shot sequences such as RARE and MPRAGE, decoupling c from acquisition parameters such as ETL. We have demonstrated that the ability to select c gives more control over image contrast, both in RARE and MPRAGE.

## Funding

The authors have nothing to report.

## Conflicts of Interest

Dr. Rydén has nothing to disclose. Dr. van Niekerk and Dr. Schauman report grants from GE Healthcare.

## Supporting information




**Data S1:** Supporting Information.

## Data Availability

An open‐source interactive dashboard of the proposed view orders, together with a Bloch simulator of the MPRAGE sequence is hosted at https://github.com/henricryden/vieworder.
