# Supplementary material for: View Order With Arbitrary Center Echoes
Source: Magn Reson Med. 2026 Jun 18;96(5):2127–39. doi: 10.1002/mrm.70482 (PMC13527268; doi:10.1002/mrm.70482)
Supplement: Supplementary file 1 [file MRM-96-2127-s001.pdf]

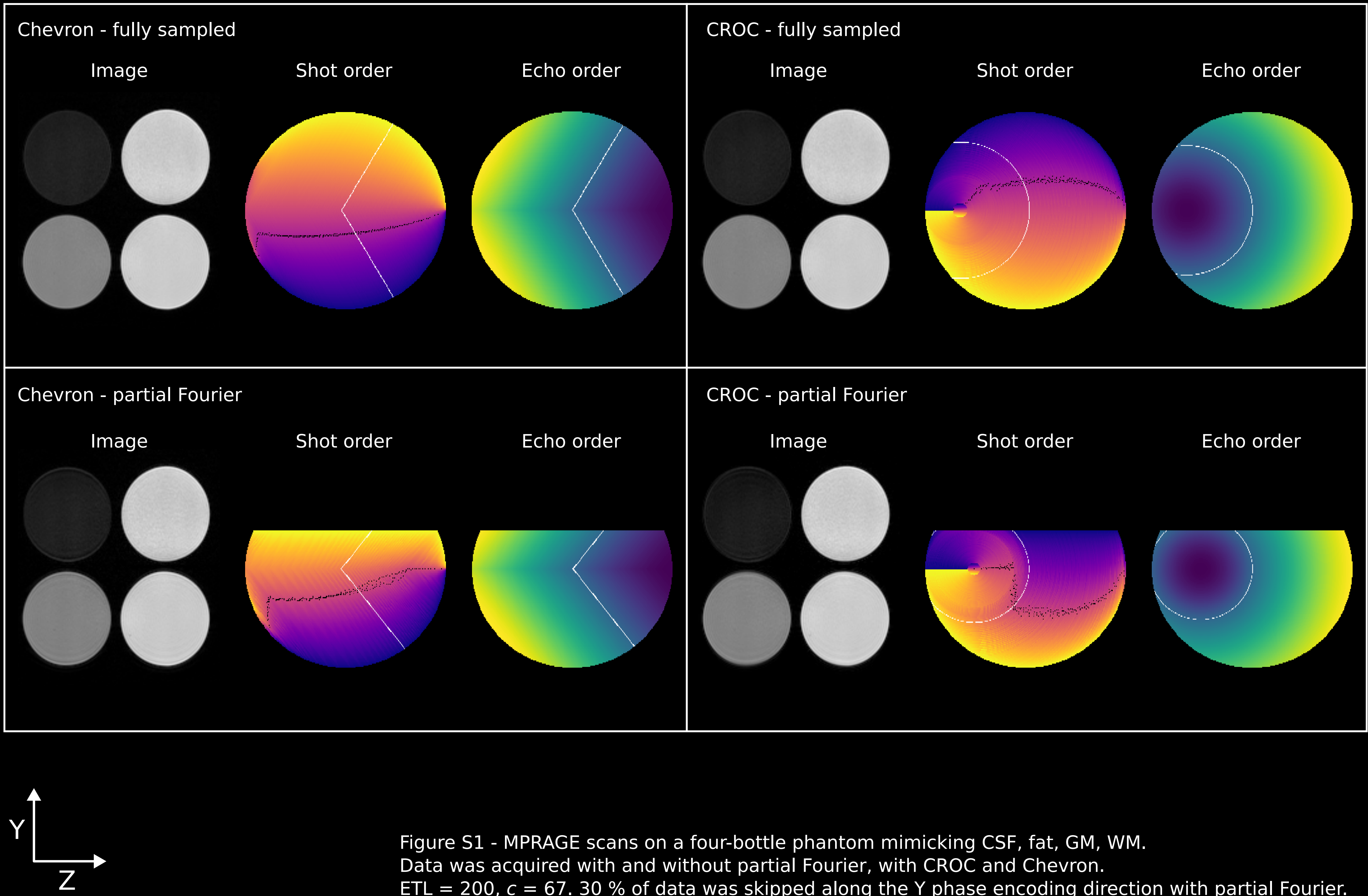

Figure S1 - MPRAGE scans on a four-bottle phantom mimicking CSF, fat, GM, WM.
Data was acquired with and without partial Fourier, with CROC and Chevron.
ETL = 200, c = 67. 30 % of data was skipped along the Y phase encoding direction with partial Fourier.
